# Plants traditionally used to make brooms in several European countries

**DOI:** 10.1186/1746-4269-3-20

**Published:** 2007-05-02

**Authors:** Anely M Nedelcheva, Yunus Dogan, Paolo Maria Guarrera

**Affiliations:** 1 Department of Botany, Faculty of Biology, Sofia University "St. Kliment Ohridski", 8, Dragan Tsankov Blvd., 1164, Sofia, Bulgaria; 2 Buca Faculty of Education, Dokuz Eylül University, 35160 Buca, Izmir, Turkey; 3 Museo Nazionale Arti e Tradizioni Popolari, Piazza Marconi 8/10 00144 Rome, Italy

## Abstract

**Background:**

The research was carried out within the course of two years (2005–2006) in four countries from southern, southeast and eastern parts of Europe: Bulgaria, Italy, Macedonia and Romania. The data are collected mainly from Bulgaria and Italy and are compared with those from Macedonia and Romania.

**Methods:**

The information was gathered largely from literature as well as field collected data and interviewed informants. A brief questionnaire, referring to the vernacular name, plant description, providing specimens from the plants and brooms, details on their use has been prepared and applied.

**Results:**

The total number of species as brooms in the study areas is about 108. The list includes two fungi taxa which caused the so-called "Witches' brooms". A high species diversity of 106 taxa of vascular plants, belonging to 37 families and 74 genera, is established in the research area. The investigation includes data about scientific name, family, vernacular name, life form, status (wild or cultivated), used parts and place of use. The relations between the plant characteristics and broom specific shape and working qualities, details of the traditionally broom planting and making, the broom as a part of folklore, traditions and religious rituals are discussed.

**Conclusion:**

Collected data show how ecological, geographical features and different cultures are related with the variety of plants traditionally used as brooms as well as details for their uses. The data about the variety of plants traditionally used to make brooms and the ways in which they are used according to the specific characteristics of the areas are important for ethnobotanical knowledge.

## Background

The interest and knowledge of plants used by native people, called ethnobotany, have increased in recent years, and there is a lot of information throughout the world [1-4]. The field of study of plants used in household products is one of the most interesting ones and it is not always easy for species to be identified.

For centuries the brooms have been used for cleaning houses, ovens, fireplaces, yards, streets, as ritual tools as well as for some special functions. For a long period (before the 18th century), brooms were domestically produced and hand-made of tree branches, brushes, etc. The broom was an important tool in keeping the living area clean. Unfortunately, dust and ashes are part of life and perfect brooms do not exist. But since ancient times people's ambition to create better and better brooms has brought rich experience of used plants and brooms. This knowledge was passed on from generation to generation and so came to us.

For economic reasons, keeping cleanness in houses, areas around houses, farmyards, streets, etc. brooms are a daily necessity. Together with the use of technical instruments for cleaning streets and yards, brooms are still in use. Planting raw material and broom manufacture continue in present days.

A broom is a cleaning tool consisting of stiff fibres attached to (and roughly parallel to) a cylindrical handle – broomstick. Some shrubs (evergreen, semi-evergreen and deciduous) from the Fabaceae family – mainly *Chamaecytisus*, *Cytisus *and *Genista *species – are commonly referred to as brooms in Western Europe. The plants belonging to these genera show similar dense aerial parts, very small leaves and slender green stems.

The Latin specific name "scoparius' means broom-like [5]. In different parts of the world, the plants used for brooms are called after the name of the relevant country or region: Spanish broom (*Spartium junceum *L., syn.*Genista juncea *(L.) Scop.), also known as Weaver's broom; Scotch broom (*Cytisus scoparius *(L.) Link); Atlas broom (or Moroccan broom or Pineapple broom) (*Argyrocytisus battandieri *(Maire) C. Raynaud, syn. *Cytisus battandieri *Maire); Provence broom or Spanish Gold Hardy Broom (*Cytisus purgans *(L.) Boiss.); Portuguese broom (*Cytisus multiflorus *(L'Hér.) Sweet), French broom (*Genista monspessulana *(L.) Johnson) etc. [1-4]. In each country or district the most frequently used plant for making brooms is called Common broom or Broom.

The known data on plants that are used as brooms is part of ethnobotanical research in different regions or countries [6-9] as well as some surveys concerning the plants traditionally used for brooms, basketry practices and plaited crafts [10-13].

The original idea that brooms from different parts of the world should be collected has been realized at Laurent's World Broom Collection since 2002 at UMMA (University of Michigan Museum of Anthropology), USA, where several samples from Europe are stored. The material used for brooms is referred mainly as "plant material" and it is not classified [14,15].

The aim of this study is to determine and introduce the plants traditionally used as brooms – which are slowly dying out-in different cultures as well as in several European countries.

## Methods

**Figure 1:**
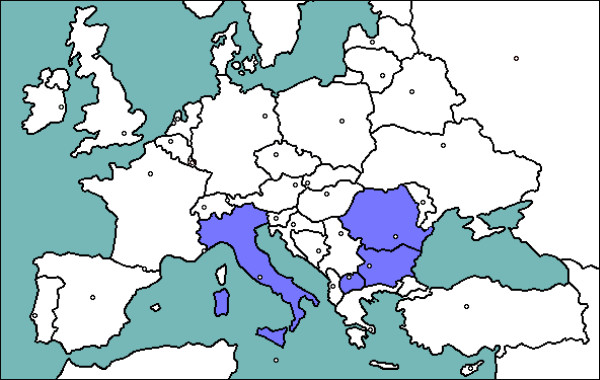
Map of the research area.

The research was carried out in four countries from southern, southeast and eastern parts of Europe. The study area range from the Italian Peninsula (that extends into the Mediterranean Sea) to the Balkan Peninsula as well as the continental parts located to the north (border on the Black Sea) (Fig. 1). Larger part of the study area includes neighbouring in geographic placement countries that have historical and cultural relations as well and mutually influence. They at one time have specific ecological conditions and national traditions. Since oldest time, their history and culture have been deeply influenced through contacts with the Mediterranean countries (as Italy). The data are collected mainly from Bulgaria and Italy and are compared with those from Macedonia and Romania. The study was carried out within the course of two years (2005–2006).

The information is gathered largely from literature (old and newest written sources) [16-45] as well as field collected data (authors' observations) and interviewed informants. Information is gathered using non-structured interviews from informants living mainly in villages, age range 38–75 years, most of them are retired farmers. A brief questionnaire, referring to the vernacular name, plant description, providing specimens from the plants and brooms, details on their use has been prepared and applied. The Bulgarian common names are given according to Field guide to the vascular plants in Bulgaria [46,47]. The plant nomenclature is given according to Flora Europaea [48].

Voucher specimens of plants (original collected data) have been deposited in the Herbarium of Sofia University (SO), Bulgaria.

## Results and discussion

### Plant diversity

The total number of species used as brooms in study areas is about 108, as some of the taxa are referred to as "spp." species plural. The list includes two (2) fungi taxa: *Melampsorella caryophyllacearum *J. Schrot and *Taphrina *spp. which caused pathogen defense structures used as brooms. A high species diversity of 106 taxa of vascular plants in the research areas is established, belonging to 37 families and 74 genera.

Due to different level and state of ethnobotanical knowledge in the countries included in the research area, great difference among them regards the number of established taxa is registered: Bulgaria (28) (Table 1), Italy (74) (Table 2), Macedonia (1) and Romania (9) (Table 3). For this reason comparative analysis of species number and diversity is not appropriate.

**Table 1:** Species established to be used as a broom in the study area: Bulgaria. Collected data – original data for current investigation.

**Taxon**	**Family**	**Vernacular name**	**References**
*Alnus incana *(L.) Moench.	Betulaceae	Bjala elsha	[46]
*Artemisia annua *L.	Asteraceae	Ednogodishen pelin	[16, 19-20, 33]
*Artemisia scoparia *W. et K.	Asteraceae	Metloviden pelin	[19-20, 46]
*Bassia scoparia *(L.) A.J. Scott	Chenopodiaceae	Obiknovena metla	[46]
*Bassia prostrata *(L.) A.J. Scott	Chenopodiaceae	Polegnala metla	Collected data Fig. 4 [SO 104396]
*Betula pendula *Roth	Betulaceae	Obiknovena breza	[46, 19-20]
*Calluna vulgaris *(L.) Hull.	Ericaceae	Obiknovena kaluna	[20]
*Caragana frutex *(L.)K. Koch	Fabaceae	Hrastovidna karagana	[19-20, 46]
*Carduus *spp.	Asteraceae	Magareshki bodil	[27]
*Carpinus orientalis *Mill.	Betulaceae	Keljav gabar	[19-20]
*Centaurea *spp.	Asteraceae	Harmanska metlichina	[16, 19-20, 28, 46]
*Cephalaria transylvanica *(L.) Roem. & Schult.	Dipsacaceae	Transilvanska zvezdoglavka	[46]
*Cirsium *spp.	Asteraceae	Palamida	[27]
*Cornus mas *L.	Cornaceae	Obiknoven drjan	[19]
*Corylus avellana *L.	Corylaceae	Obiknovena leska	[19]
*Cytisus agnipilus *Velen.	Fabaceae	Rodopski zanovetz	[20]
*Erica arborea *L.	Ericaceae	Strandzhanski piren	[19-20, 46]
*Erica manipuliflora *Salisb.	Ericaceae	Piren	[19]
*Melampsorella caryophyllacearum *J. Schrot. (it causes gall of stem of *Abies *spp.)	Uredinales	Samodivski metli, Veshtichni metli	Collected data Fig. 5 [SO 104397]
*Panicum miliaceum *L.	Poaceae	Tatarsko proso	[27]
*Phillyrea latifolia *L.	Oleaceae	Ggripa	[20]
*Salix *spp.	Salicaceae	Varba, Rakit	[19]
*Scabiosa ochroleuca *L.	Dipsacaceae	Zhaltenikava samogrizka	[46]
*Sorghum bicolor *(L.) Moench.	Poaceae	Metla, sorgo	[19, 46], Fig. 7–9
*Spiraea salicifolia *L.	Rosaceae	Varbolisten tazhnik	[20]
*Spiraea media *F. Schmidt.	Rosaceae	Tazhnik	Collected data [SO 104398]
*Taphrina *spp. infectious agent on *Prunus*, *Betula*, *Carpinus *spp.	Ascomycetes	"Rozhkovi"	Collected data Fig. 6
*Xeranthemum annuum *L.	Asteraceae	Obiknoveno bezsmartniche	[16, 46]

**Table 2:** Species established to be used as a broom in the study area: Italy

**Taxon**	**Family**	**Vernacular name**	**References**
*Ammi visnaga *(L.) Lam.	Apiaceae	Tsikkirìa biànka	[22]
*Ampelodesmos mauritanicus *(Poiret) T. Dur. et Sch.	Poaceae	Tagliamani, erba alfa, jaccole (Basilicata)	[22, 26, 29]
*Apium graveolens *L.	Apiaceae	Sedano	[22]
*Arum maculatum *L.	Araceae	-	[22]
*Arundo donax *L.	Poaceae	Canna	[22, 26]
*Arundo plinii*Turra	Poaceae	Cannìccia (Marche)	[26, 30]
*Asparagus *spp.	Liliaceae	Skòvas de axròba, skovàtsus	[22]
*Asphodelus *spp.	Liliaceae	Asfodelo	[22]
*Ballota nigra *L.	Lamiaceae	-	[22]
*Bassia scoparia *(L.) A.J. Scott	Chenopodiaceae	Skova de fòrru	[22]
*Betula pendula *Roth.	Betulaceae	Biulla	[31]
*Calicotome villosa *(Poir.) Link	Fabaceae	-	[22]
*Chamaerops humilis *L.	Palmae	Palma nana, margagliò	[22]
*Chrysanthemum coronarium *L.	Asteraceae	Karagàntsu	[22]
*Chrysanthemum segetum *L.	Asteraceae	Karagàntsu	[22]
*Chrysopogon gryllus *(L.) Trin.	Poaceae	Tribi	[31]
*Cistus monspeliensis *L.	Cistaceae	-	[22]
*Cistus *spp.	Cistaceae	-	[22]
*Conium maculatum *L.	Apiaceae	Feruledda	[22]
*Cornus mas *L.	Cornaceae	Sanginello	[26]
*Cyperus longus *L.	Cyperaceae	Giùnku fèmmina, séssini	[22]
*Cytisus scoparius *(L.) Link	Fabaceae	Ginestra, scopa, scopiglio, maggio, jnèstra	[18, 23, 31-33]
*Daphne gnidium *L.	Thymelaeaceae	Su truìscu	[22]
*Dittrichia viscosa (*L.)Greuter subsp. *viscosa*	Asteraceae	Fitintusa, pulicara (Sicily), crisi (Basilicata)	[22, 26, 39-40]
*Ephedra major *Host	Ephedraceae	-	[22]
*Erica arborea *L.	Ericaceae	Elica masculina (Basilicata), scopinello, (Latium), skòva, iskòpa (Sardinia)	[18, 22-23, 26, 34-35]
*Erica herbacea *L.	Ericaceae	Brüf	[31]
*Erica multiflora *L.	Ericaceae	Elica femminina	[26]
*Erica scoparia *L.	Ericaceae	Scopuccio (Latium), skòva, iskòba, iskòpa (Sardinia)	[22]
*Erica terminalis *Salisb.	Ericaceae	Skòva, i skòba, iskòpa	[22]
*Euphorbia characias *L.	Euphorbiaceae	Erba mora (Latium), pede de lupo (Basilicata)	[18, 26]
*Ferula communis *L.	Apiaceae	-	[22]
*Foeniculum vulgare *Miller	Apiaceae	Finòcchju	[22]
*Genista aetnensis *(Raf. ex Biv.) DC.	Fabaceae	-	[22]
*Genista monspessulana *(L.) L. Johnson	Fabaceae	-	[22]
*Genista radiata *(L.) Scop.	Fabaceae	Spazzarìne, spazzole	[36]
*Genista thyrrhena *Valsecchi	Fabaceae	Fascina	[37]
*Helichrysum italicum *(Roth) G. Don fil	Asteraceae	Skòva de sànta Maria (Sardinia)	[22, 34]
*Helleborus foetidus *L.	Ranunculaceae	Scopacci	[38]
*Ilex aquifolium *L.	Aquifoliaceae	Trentavècchie	[18]
*Juniperus phoenicea *L.	Cupressaceae	-	[22]
*Laurus nobilis *L.	Lauraceae	Lauro	[26]
*Malva sylvestris *L.	Malvaceae	-	[22]
*Marrubium vulgare *L.	Lamiaceae	-	[22]
*Myrtus communis *L.	Myrtaceae	Murtìdda	[22, 26]
*Nicotiana glauca *Graham	Solanaceae	-	[22]
*Olea europaea *L. subsp. *oleaster *(Hoffm. & Link.) Negodi	Oleaceae	Acebuche; olibondo basatia	[22]
*Oryza sativa *L.	Poaceae	-	[22]
*Ostrya carpinifolia *Scop.	Betulaceae	Càrpeno	[41, 43]
*Osyris alba *L.	Santalaceae	Scannagad-dìne (Basilicata); skòva de bìnga, skoviòi (Sardinia), curisurgi (Sicily)	[22, 24, 26]
*Phragmites australis *(Cav.) Trin. ex Steud.	Poaceae	Kannisòni (Sardinia), cannarìzze, cannucce (Abruzzo)	[22]
*Philyrea angustifolia *L.	Oleaceae		[22]
*Pistacia lentiscus *L.	Anacardiaceae	Stìnku, listìnku	[22]
*Prunus spinosa *L.	Rosaceae	-	[22]
*Pteridium aquilinum *(L.) Kuhn	Hypolepidaceae	Filici	[26, 39]
*Rubus ulmifolius *Schott	Rosaceae	Rogo, spine	[23]
*Ruscus aculeatus *L.	Liliaceae	Piccasorce, ruscio	[22, 26]
*Salix alba *L.	Salicaceae	Salcio	[17]
*Salix *spp.	Salicaceae	-	[22]
*Sambucus ebulus *L.	Caprifoliaceae	Iévulu	[18, 26]
*Sambucus nigra *L.	Caprifoliaceae	-	[22]
*Scabiosa atropurpurea *L.	Dipsacaceae	'Nfilamura	[24]
*Smyrnium olusatrum *L.	Apiaceae	-	[22]
*Sorghum *bicolor (L.) Moench.	Poaceae	Melica, saggina (Central Italy), saìna de skòvas (Sardinia)	[18, 22-23, 29, 41-44]
*Spartium junceum *L.	Fabaceae	Ginestra (Marche), spartu (Basilicata)	[26, 30, 36]
*Stachys glutinosa *L.	Labiatae	Skòva de argòas, munda dòmos	[22]
*Styrax officinalis *L.	Styracaceae	Ciucciupìcchiu, armella	[18]
*Thapsia garganica *L.	Apiaceae	Skòva de fòrru	[22]
*Thymelaea hirsuta *(L.) Endl.	Thymelaeaceae	Nerbiàtsu, vorru	[22]
*Urtica urens *L.	Urticaceae	-	[22]
*Urtica *spp.	Urticaceae	-	[22]
*Verbascum pulverulentum *Vill.	Scrophulariaceae	-	[22]
*Verbascum thapsus *L.	Scrophulariaceae	Kadùmbu	[22]
*Vitis vinifera *L.	Vitaceae	-	[22]

**Table 3:** Species established to be used as a broom in the study area: Macedonia and Romania. Collected data – original data for current investigation.

**Taxon**	**Family**	**Vernacular name**	**References**
**Macedonia**			
*Bassia scoparia *(L.) A.J. Scott	Chenopodiaceae	Tarla süpürge	Collected data Fig. 10
**Romania**			
*Alnus glutinosa *(L.) Gaertn.	Betulaceae	Arin	Collected data
*Bassia scoparia *(L.) A.J. Scott	Chenopodiaceae	maturi	Collected data
*Betula pendula *Roth.	Betulaceae	mesteacan	Collected data Fig. 11
*Carpinus betulus *L.	Corylaceae	carpen	Collected data
*Prunus spinosa *L.	Rosaceae	porumbar	Collected data
*Salix capraea *L.	Salicaceae	Salcie/capreasca	Collected data
*Salix purpurea *L.	Salicaceae	Salcie/capreasca	Collected data
*Salix rigida *Muhl.	Salicaceae	Salcie/capreasca	Collected data
*Sorghum bicolor *(L.) Moench.	Poaceae	Sorg/maturi	Collected data

**Figure 2:**
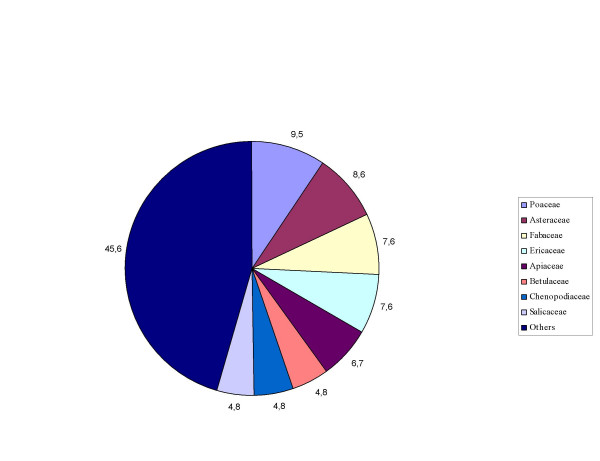
Systematic structure of the established taxa [%].

Most of the plants, with the exception of *Pteridium aquilinum *(Pteridophyta), are representative of Magnoliophyta. The participation of Gymnosperms (5.4%) is insignificant. Monocotyledonae are present with 5 families (13.5%) and Dicotyledonae with 29 families (78.4%). The predominant number of species is from Poaceae (10), Asteraceae (9), Fabaceae (8), Ericaceae (9), Apiaceae (7), Betulaceae (5), Chenopodiaceae (5) and Salicaceae (5). The rest of the families contain 1–4 species (Fig. 2).

**Figure 3:**
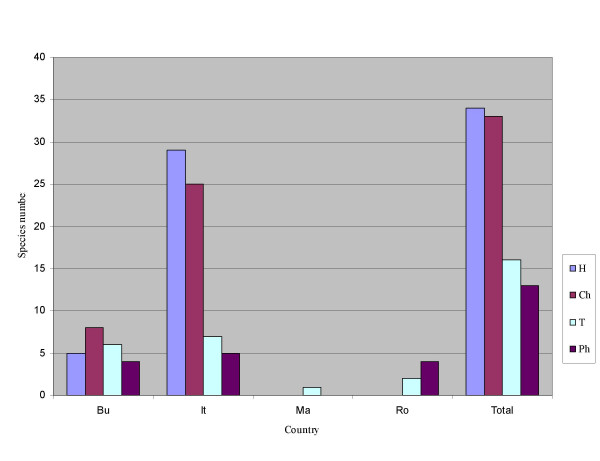
**Species life forms structure in different parts of the study area**. Countries: Bu (Bulgaria), It (Italy), Ma (Macedonia) and Ro (Romania); Life forms: Ph: phanerophyte; Ch: chamaephyte; H: hemicriptophyte; T: therophyte.

Hemicryptophytes species prevail (34), followed by chamaephytes (33) (evergreen shrubs (17) and shrubs (16)), therophytes (16) and phanerophytes (13) (Fig. 3). The structure of the life forms of the established species is according to the research areas' specific conditions. On the other hand the life form's structure corresponds to the plant's diversity with features suitable for certain broom qualities – strength and flexibility.

**Figure 4:**
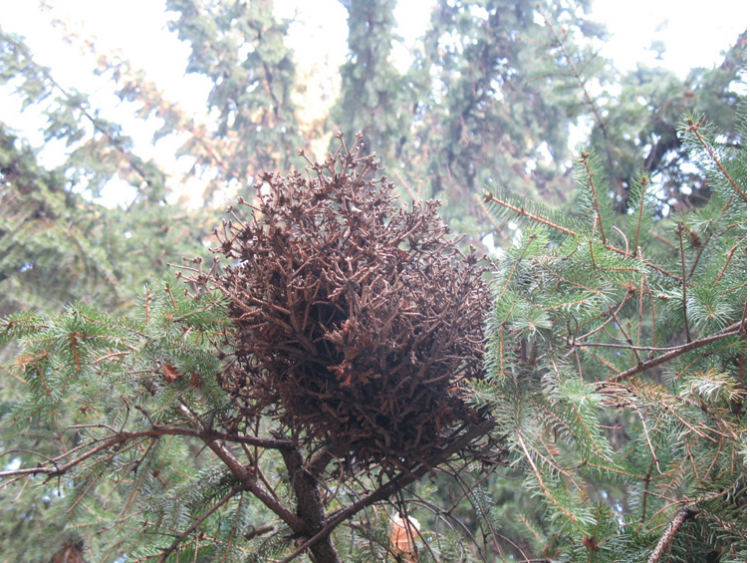
Witches' broom on *Picea abies*, Kyustendil, Bulgaria.

**Figure 5:**
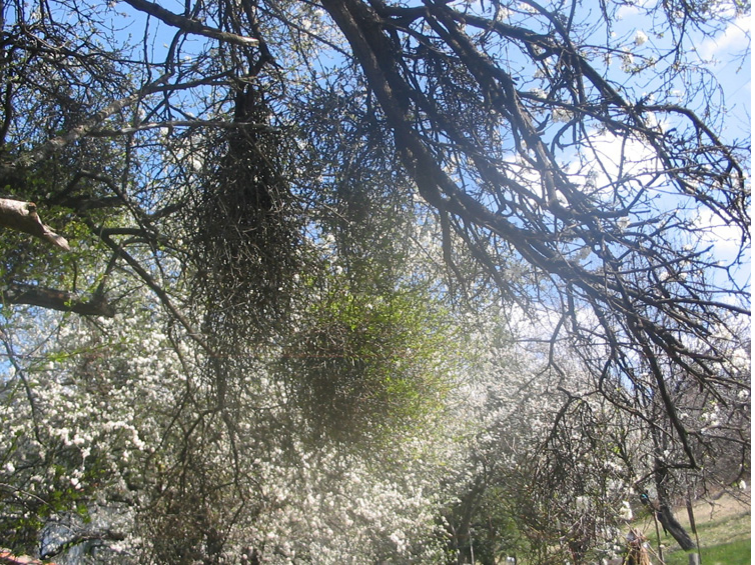
Witches' broom on *Prunus cerasifera*, village Poletintzi, Bulgaria.

Witches' brooms are a special case. They are deformities on trees or shrubs as a result of infections by different agents. The rates of plant growth, size and symmetry are disrupted. A certain part of the plant may look like a bird's nest (closely packed distorted twigs) (Figs. 4, 5). These formations are very suitable in terms of shape, strength and flexibility for broom material [25]. In the past, mysterious and unexplainable occurrences were often blamed on witchcraft. Witches' brooms occur on many different woody plant species, including deciduous trees such as hackberry, maple, and willow, and conifers such as pine and spruce. At first, only the appearance of the shrub is affected, but continued feeding over a period of years often stunts the honeysuckle's growth, eventually weakening it and contributing to the shrub's death [25]. Most of the species are wild plants (78.4%). Some of them are cultivated for different needs (10.8%) as food and ornamental, except *Sorghum bicolor *and *Bassia scoparia *which are cultivated only for making brooms. The main plant cultivated for brooms in the study area is *Sorghum bicolor *(on small areas). In Bulgaria the area for *Sorghum *cultivation never had exceeded thirty five thousand decare. One part of species is wild, but they meet together as well in a culture in the same area (9%). In Italy, the use of wild plants for making brooms is more common as a result of plant richness and national traditions. In other parts of study area the use of cultivated plants is wider.

The most mentioned plants to be used as brooms in study area are *Bassia scoparia*, *Sorghum bicolor*, *Betula pendula*, *Cornus mas *and *Salix *spp. (Bu, It, Ma and Ro). The most common wild plants that are used for broom making in Bulgaria and Italy include: *Erica *spp. and *Cytisus *spp. Some plants used in Italy are similar to known data for Spain, Portugal and Turkey, according to Mediterranean ecological conditions, as well *Erica scoparia*, *Cytisus scoparius*, *Marrubium vulgare*, *Olea europaea*, *Verbascum *spp., *Cistus *spp., *Genista *spp., *Phillyrea angustifolia*, *Osyris alba*, and etc. [10-13].

The registered species *Bassia prostrata*, *Spiraea media *and witches' brooms (caused by *Melampsorella caryophyllacearum and Taphrina *spp.) have not been referred before for the Bulgaria, according to the references (Table 1).

### Ethymology (history of the names)

"M е т Л а" (metla) is the Bulgarian name for broom. The diminutive name is "м е т Л и ч k а" (metlichka), often used for smaller ones and in folklore tales and folklore songs, this is a proof of its great importance in people's daily customs and its position in their life. "Metlar" is used for broom-maker or broom-seller. Many plants used as brooms are called "metla" in Bulgarian; so are plants whose shape is similar to that of a broom (*Asparagus *spp., *Poa *spp., *Lactuca *spp., etc.) [16].

In central-southern Italy peculiar brooms used for sweeping farmyards are called "granate", see e.g. *Salix alba *[17]. In the National Museum of Arts and Folk Traditions a broom ("granata") from Apulia region, probably made of *Thymus capitatus*, is kept.

Several plants in Italy are called with vernacular names that mean "broom": e.g. *Bassia scoparia*, "skova de fòrru"; *Asparagus *spp. "skòvas de axròba, skovàtsus"; *Cytisus scoparius*, "scopa, scopìglio"; *Erica arborea*, "scopinello, iskòpa"; *Erica scoparia*, "scopuccio" etc.; *Genista radiata*, "spazzole"; *Helichrysum italicum*, "skòva de Santa Maria"; *Helleborus foetidus*, "scopacci"; *Thapsia garganica*, "skòva de forru" etc.

In some cases the vernacular name comes directly from Latin language, for example: "munda domos" in Sardinia (*Stachys glutinosa*), because in Latin language: mundare = to clean; domus = house.

In central Italy the little broom for cleaning ovens is called "mùnnulo": it is also made of some toxic plants, e.g. *Helleborus foetidus *and *Sambucus ebulus *[18].

### Broom making craft

Making a broom is more than a plain activity, it is an art. Broom makers are familiar with broomcorn (morphological features, phenology, phytopathology, etc.). The observations gained on a plant are subsequently used in the production of brooms.

**Figure 6:**
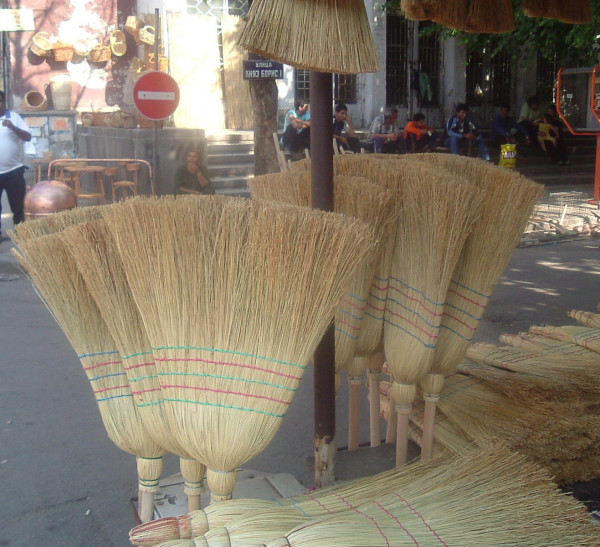
Brooms from *Sorghum bicolor *in the street market in Sofia, Bulgaria.

**Figure 7:**
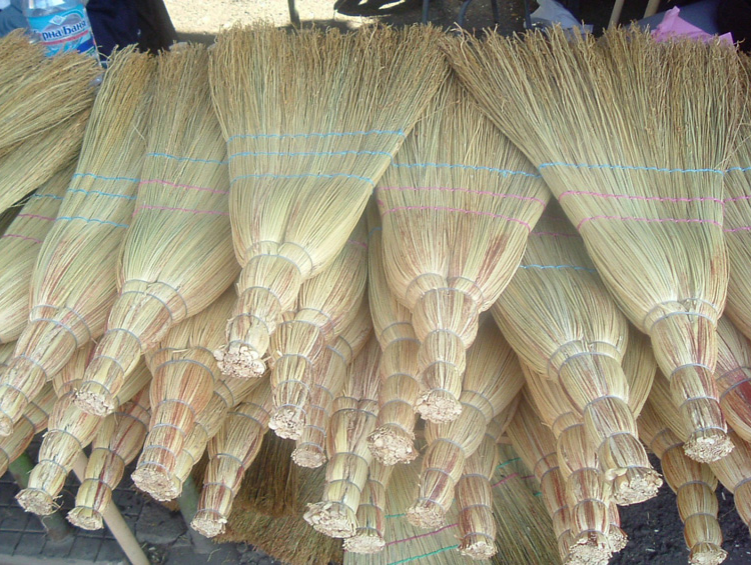
Industrial manufactured brooms from *Sorghum bicolor *in the street market in Sofia.

**Figure 8:**
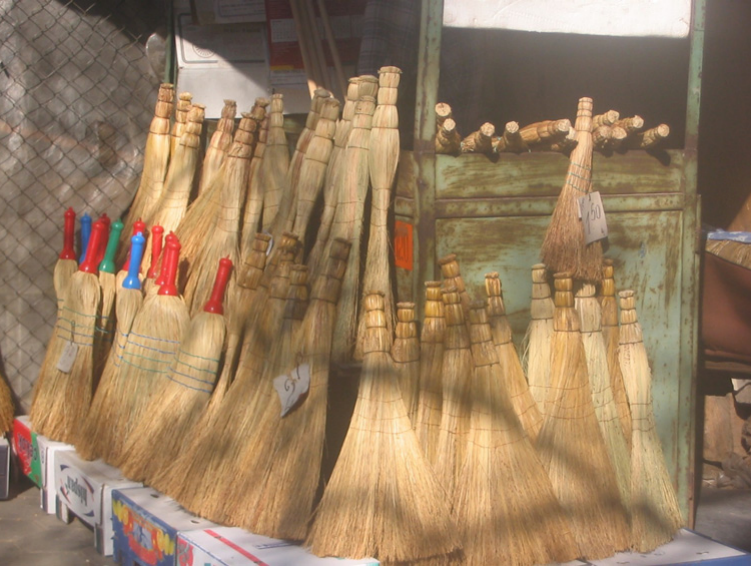
Manufactured brooms from *Sorghum bicolor *in the street market in Kyustendil.

*Sorghum bicolor *is used for industrial manufacture only (Figs. 6, 7, 8). The part used is the panicle. In Bulgaria, it is miscalled "metlov klas" (panicle ear) in practice. The same name is used in some official documents. The panicle branches are called "zhitzi" (wires). Some broom-makers break the panicle stem (axis) under the first internodes, this causes faster blossoming and seed maturing – which results in better quality panicles for brooms. The gathering of the panicles with mature seeds determines the typical colour of the glumes (in the first half of September). People know that reddish colour of panicles is caused by plant louses (*Aphis *sp.) or that it is the natural colour of some varieties, brownish is a result of fungi diseases, etc. [19].

The panicles without seeds are dried in open, sunny places (commonly in yards). After drying, panicles are grouped and bound together in small sheaves with hemp cord, fiber, mulberry branches or metal wire.

The remaining stems are used for firewood and for making temporary roofs. Broomcorn produces a large amount of plant material. Stems and folia are used as forage for sheep and cattle. The seeds are good food for cattle and birds [19-21].

The making of broom crafts is a men prerogative (rarely women). Also gypsies are known as good broom makers.

The region with the oldest traditions in broom planting and making (more than 200 years) is the village of Michaltzi (Veliko Tarnovo district, Bulgaria). In the middle of last century people called the village "Metlen – burg" (Broom-castle) as an analogue to some German towns, with typical Bulgarian sense of humour. The field work-free time is used – especially in winter – for broom making. The prepared brooms are used for barter; so are eggs in other villages. Six kinds of brooms from this region are known – common house broom, parson's broom, double broom, bettered "mihalska" broom, broom for dresses, attic broom. Models from foreign countries – Russia, Italy and Philadelphia – are imported for trade [19].

**Figure 9:**
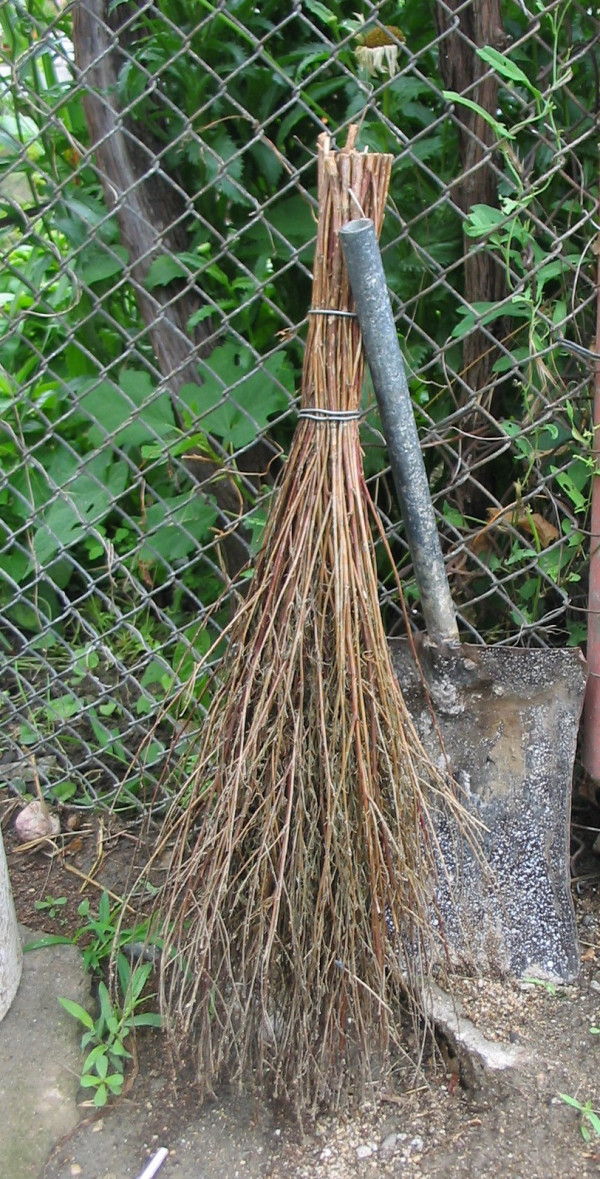
Broom of *Bassia prostrata *– Struma valley, village Lebnitsa, Bulgaria.

**Figure 10:**
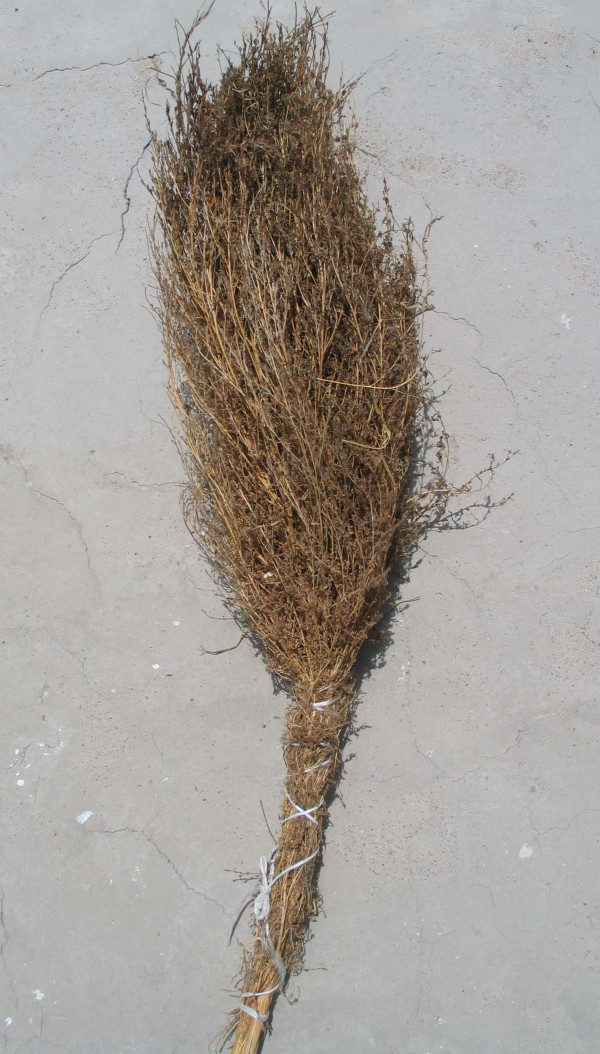
Broom from *Bassia scoparia*, Macedonia.

**Figure 11:**
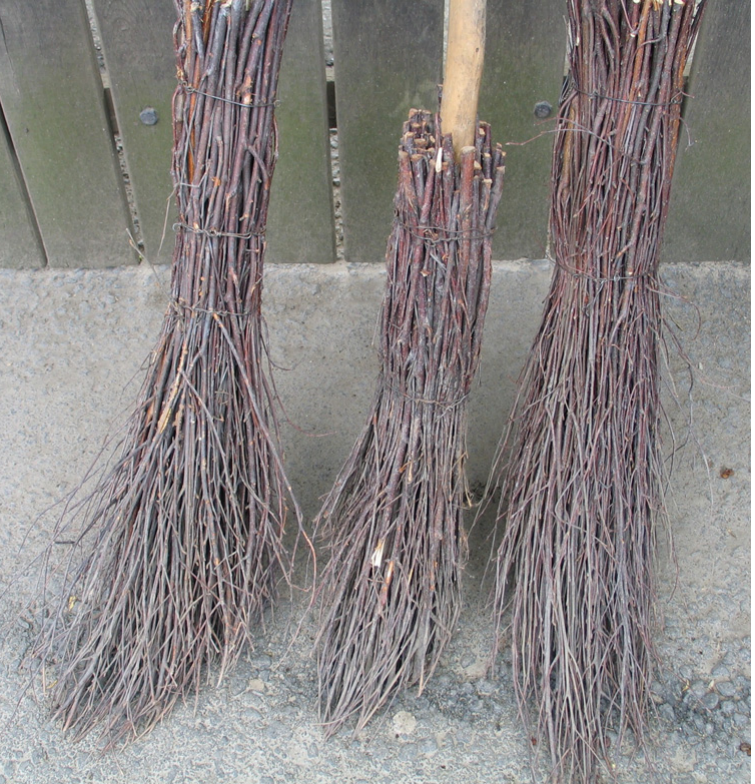
Brooms from *Betula pendula*, Romania.

Well branched shrubby plants with strong, sinewy fibers and flexible twigs are selected for broom making (Ericaceae, Betulaceae, Fabaceae, Asteraceae, Rosaceae, Chenopodiaceae, Betulaceae, etc.) [19]. Making shrubby brooms is simple. The stems are bound together with sinewy wands, twisting plant stems or fiber around them while still fresh (Figs. 9, 10, 11). They are used after drying [19]. But in many cases a raw plant is used for shrubby brooms, it is available, and its technical quality is of no importance. These brooms are for private use, not for trade. This kind of brooms is typical of very poor and remote villages and districts.

Some kind of brooms for domestic and industrial use has different kinds of wooden handles (made of beech wood and linden, untreated beech wood or hornbeam wood).

In Sardinia, Italy, *Arundo donax *and *Juniperus phoenicea *stems are often used as handle respectively for *Chamaerops humilis *and *Cistus monspeliensis *brooms [22].

Some plants with strong fibres are locally used for binding: in Latium brooms made of *Sorghum bicolor *are bound with *Rubus ulmifolius *stem [23], while in Sicily *Scabiosa atropurpurea *stems are tied with *Ampelodesmos mauritanicus *leaves [24].

Brooms have a specific shape and working qualities. For these characteristics to be obtained different parts of the plants are used e.g.: stem (*Genista radiata*), branches (*Salix alba*), leaves (*Chamaerops humilis*), panicle (*Sorghum bicolor*) or aerial parts (*Erica arborea*).

In the past, plants of brooms have been growing at each house. Later, an one or several humans in the village or the region start to cultivate and make brooms, as a way to living. Small production workshops and manufactures are established gradually. Such more modern factories are present in nowadays. Their production comprises a wide range of straw brooms and brushes (for domestic and industrial use), different kinds of wooden broomsticks and details according to customers' wish. They export large quantities of the production (e.g. Greece) [19].

### Broom uses

Local folk classifications (folk nomenclature) are result of daily experience of people using brooms. They called them soft and hard brooms, big and small brooms, depending on the type of plant they are made, their size and use. Names also are given according to their use (yard broom, home broom, threshing-floor broom, etc.) [16,19-21,46].

Some plants are used for making crude shrubby brooms, e.g. species of the Ericaceae, Betulaceae, Fabaceae etc. families, for cleaning streets and yards (including village square, farmyard, stack-yard and threshing yard, sheds, roads, all open areas, etc.).

Soft brooms from shrubs or herbs (Asteraceae, Lamiaceae, Poaceae, Liliaceae, Apiaceae, etc.) are used to clean several places inside the house as threshing floor, cellar, kitchen, oven, fire place, to clean pots, etc.)

Particular brooms were made of sticky plant parts: e.g. in Sardinia of *Stachys glutinosa *[22] or of repellent and sticky materials: e.g. in Basilicata of *Dittrichia viscosa *to remove fleas [26]. In order to eliminate thorns from *Opuntia ficus-indica *fruits were used *Vitis vinifera *shoots and *Dittrichia viscosa *leafy branches [22].

*Ruscus aculeatus *branches are commonly used for cleaning chimneys and walls from cobwebs [22].

Cleaning with brooms made from aromatic plants take the "burned food" odour away from ovens and stove burners, while oven and burners are still hot. In the research area are established 28 species used for special in ovens and fire places (all of them from Italy) – 9 of them are aromatic plants (*Apium graveolens*, *Foeniculum vulgare*, *Ferula communis*, *Nicotiana glauca*, *Laurus nobilis*, *Myrtus communis*, *Pistacia lentiscus*, *Ballota nigra *and *Marrubium vulgare*) as well some toxic plants as *Conium maculatum *and *Daphne gnidium*. For *Ferula communis*, *Malva sylvestris *and *Nicotiana glauca *is mentioned special use in ovens for bread [22,26]. For the other studied countries such data was not found.

Many broom species are widely used as ornamental landscape plants (*Calluna vulgaris*, *Erica arborea*, *Spiraea media*, *Spiraea salicifolia*, *Cytisus scoparius*, *Laurus nobilis *etc.) as well as for wasteland reclamation and sand dune stabilizing. Others are popular in horticulture and many of them are cultivated hybrids like Kew broom (*Cytisus *× *kewensis*) and Warminster broom (*Cytisus *× *praecox*).

Some "common broom" species, introduced as ornamental plants or cultivated for broom-manufacturing, became naturalized and invasive weeds because of their aggressive dispersal seed.

Cleaning with a broom of some plant species is part of many folklore traditions and religious rituals. Brooms of *Centaurea *spp. are used at home and are objects of beliefs; they have a special place in the home [20,21,27,28,46,49]. The plants are collect in special day of the summer named in Bulgarian traditions "Enyovden" (24 June). The made by them brooms, are guarded in the house until the next year. This broom is kept to the fire-side or behind the door, to protect the family and the family from illnesses and evils. Through the spring the older broom sets on fire and throws on the waste. The fleas, the snakes and the lizards from the house are chased so away. A broom is not to be jumped, it is sin. If a girl is beaten with a broom, she will not marry. A broom if a little child is being left gone merely in a room is put next to it to defend it against an evil spirits. The broom is used for human treatment by fear. Through the spring customs a broom from the fire-side is stolen. The more the hostess is anger, that more rains there are. Later, the same broom draped in rags, and the stick is formed as the head with the eyes, eyebrows and a mouth. The name of this human's figure is "German". But this one "German" is used in rituals for drought, against rain [49].

A Basil (*Ocimum basilicum*) broom plays a special role in folklore tales and songs [21,28,49]. In Italy (and other several European countries) people believed that a broom made with *Sorghum bicolor *or with other plants, put behind the door of the house, could take away the witches (witches, counting the stem, made mistakes; they were occupied along the entire night counting the stems, therefore they do not could entry in the house [21,27,28].

## Conclusion

As a result high species diversity of vascular plants in the research areas is established. Collected data, no matter what the level of ethnobotanical knowledge in different parts of the study area, shows how ecological, geographical features and different cultures are related with the variety of plants traditionally used as brooms as well as details for their uses.

What are the historic roots of the use of plants for brooms and of broom making craft? This is one of the questions that cannot have only one correct answer. Studies, as presented here throw light on this field.

Nowadays, the use of home-made brooms and broom making craft are disappearing. The brooms have lost their necessity in our daily lives. The principal reasons are realities of the modern life: loss of the specific tasks and places which they were used, industrialization of traditional farming, the modern tools for cleaning, partial adoption of modern material, the reduced number of craftsman and etc. The skills transmission through generations is in danger and much more threatened with extinction.

The data about the variety of plants traditionally used to make brooms and the ways in which they are used according to the specific characteristics of the areas are important for ethnobotanical knowledge.

They contribute to preserving the world's human traditional experience as well as national identity.

## Competing interests

The author(s) declare that they have no competing interests.

## Authors' contributions

The work for data collection was carried out above all by research area as follow: Anely Nedelcheva by Bulgaria, Paolo Maria Guarrera by Italy and Yunus Dogan by Macedonia and Romania. Data analysis and manuscript preparation were conducted equally by authors. All authors read and approved the final manuscript.
